# Supplementary material for: Are carbon emissions trading and green financial instruments synergistic? -Comprehensive quantitative research based on content analysis
Source: PLoS One. 2024 Mar 7;19(3):e0298601. doi: 10.1371/journal.pone.0298601 (PMC10919601; doi:10.1371/journal.pone.0298601)
Supplement: S1 Data — (ZIP) [file pone.0298601.s001.zip › Data/地级市Z_F工作报告.zip!/║┌┴·╜¡/║┌║╙╩╨/└┤╫╘╣1⁄2╓┌║┼í╛┬φ┐╦╩2╛▌═°í┐_2005.docx]

各位代表：

现在，我代表市人民政府，向大会报告工作，请予审议，并请各位政协委员和列席会议的同志提出意见。

一、2004年工作回顾

过去一年，在省委、省政府和市委正确领导下，紧紧依靠全市人民，抢抓机遇，团结拼搏，开拓进取，较好地完成了市三届人大三次会议确定的任务。地区生产总值实现112.3亿元，同比增长11.6%。城镇固定资产投资完成25.7亿元，增长16.3%。社会消费品零售总额实现30.2亿元，增长10%。财政一般预算收入2.86亿元，增长10%。

经济结构调整不断深化。全市一二三次产业比例由32.8：21.6：47.6调整为35.6：20.7：43.7。农业克服多种灾害，实现增加值24.9亿元，增长37.9%。播种面积1098.9万亩，粮食总产147.4万吨，增长113.6%。“六大经济”取得明显进展。大豆总产达109.3万吨。绿色食品产值6.28亿元。畜牧业产值占农业总产值比重达到17.2%。完成造林28.6万亩，国家重点生态林管护取得成效。输出农村劳动力11万人，实现劳务收入3.5亿元。对俄境外农业种植面积2.8万亩，产值1200万元。规模以上产业化龙头企业发展到29家，组建了10个农机作业合作社。工业经济质量效益同步增长，全市规模以上工业企业实现增加值5.78亿元，增长15.5%。工业经济综合效益指数65.5。完成18个改造项目,增强了发展后劲。第三产业保持持续快速增长，实现增加值49.1亿元，增长7.3%。共接待国内外旅游者81万人次，增长61.1%，其中边境游19.5万人次，增长74.1%。五大连池被评为世界地质公园，景区AAAA级质量等级评定通过省级验收。山口湖景区、瑷珲历史陈列馆分别通过AA级、AAA级质量等级评定。黑河旅游接待中心投入使用。民营经济完成增加值19.6亿元，增长15.2%。

对外开放取得新进展。对外贸易进出口总值实现2.89亿美元，增长165.1%，外资到位654万美元。赴俄开展森林采伐、农业种植等合作项目取得新进展。成功引进了黑河太阳经贸有限公司等企业33户，进出口总值超百万边贸企业达34家，超千万企业4家。大黑河岛互贸区顺利延伸到市区，俄持简化手续入境人数达12.8万人次，同比增长317%。民贸进出口实现12527万美元，同比增长388%。逊克互贸区得到省政府批准。首次举行了黑河与阿穆尔州经贸合作推进会。实行了客货分流，通关速度明显加快。招商引资实现重大突破，共签约141项，合同总金额25.5亿元。落实项目134项，引进到位资金7.1亿元，增长39.9%。其中超千万元项目17项，增长76.1%。

各项改革顺利推进。国企产权制度改革面达95%。完成社保并轨试点任务2.1万人，为省下达计划的163%，争取国家补助资金6300余万元。全面推进了农村税费改革，全市免征农业税13984万元，发放粮食补贴资金18719万元、良种补贴资金1220万元，农民人均减负356元。粮食购销体制改革顺利推进。国库集中支付改革全面展开，启动了各县（市）区部门预算编制改革试点。清理整顿了市直事业单位，市直政府机构改革和全市乡镇精简行政编制工作顺利展开。

重点项目建设成效显著。黑龙江大桥前期工作加快推进，绥北、孙逊、沾五公路及黑河至逊克、嫩江至临江、北安通北至主星公路建成通车，农村公路通达工程完成414.6公里，新增通公路村47个。新兴基础原材料加工区实现对俄购电，一期工程6台炉投产。黑河龙珠远东国际滑雪场投入使用。象山水库除险加固工程已列入国家第二批水库除险加固规划。二门山水库复建项目已完成主体工程70%。嫩江县热电厂项目前期工作已基本完成。吉斯达物流中心基础设施建设继续推进。多宝山铜矿、东安岩金矿等13个项目进展顺利。

城市建设步伐加快。全市新开工建筑面积69.7万平方米，增长15%，其中市区35.8万平方米，创历史新高。全市新建扩建道路8.3万平方米，给排水管线13.5公里。市区完成65%街路的改造任务。完成了黑河市近期建设规划、黑龙江大桥桥头区总体规划编制。全市土地收益1.04亿元，同比增长32%，其中市本级土地收益6300万元，增长50%。生活垃圾袋装化管理启动运行。小区供热并入集中供热面积21万平方米，城市空气质量达到二级质量天数305天。化学耗氧量、烟尘、二氧化硫年排放量、集中式水源地水质达标率、工业固体废物处理率均达到省要求。

社会事业蓬勃发展。实施省市级科技计划项目32项。齐大黑河分校晋升为黑河学院。初中生辍学率控制在3%以下。高中阶段毛入学率达38%。完成中小学危房改造项目25个，新建校舍3.79万平方米。全市96%的乡镇以上中小学开设了信息技术课。村村大学生计划首批学生已分配到村屯。疾病预防控制和医疗救治体系不断完善，全市6个疾控中心项目、市120急救中心、市第一人民医院住院部大楼投入使用，6个传染病区建设项目进展顺利。成功举办了全市第三届田径运动会和第二十届《黑河之夏》文艺调演。黑河人民艺术剧院投入使用，黑河中心图书馆建设基本完成。“北三县”城域有线电视网光缆化改造完成。全市人口自然增长率控制在3.9‰。加强了征兵、安置、优抚和双拥工作，军政、军民关系更加密切。民族、宗教、外事、侨务、老龄、残疾人、审计、统计、档案、气象、文博图、地方志等工作都取得了新成绩。

人民生活条件明显改善。城镇新增就业16698人，城镇登记失业率控制在4%以下。职工工资和三条保障线资金发放率均为100%。城镇人均可支配收入4255元，增长10%。农民人均纯收入2740元，增长49.6%，达到历史最好水平。居民消费价格指数为104.5，居民储蓄存款余额107.9亿元，比年初增长3.1%。黑河市区人均住房建筑面积达到24.2平方米，增长10.5%。人均公共绿地面积达5.38平方米，增长18%。村镇人均住房建筑面积达到18.4平方米，增长2%，住房砖瓦化率达到60.8%，自来水普及率达96.5%。

社会稳定工作态势良好。广泛开展赈灾济困活动，发放农村特困救助证1.6万个、灾民救助卡3.86万个，救助困难群众12.9万人次。认真调处信访案件，维护了政治安定和社会稳定。大力推进社会治安综合治理，集中处理了一批长期积压的大案要案。加强煤矿、交通、消防安全管理，安全生产形势总体平稳。

政府自身建设进一步加强。自觉接受市委领导和人大、政协监督，定期报告工作和通报情况。办理人大代表建议46件，政协委员提案194件，办结率均为100 %。加强与工会、共青团和妇联等群众团体的联系，广泛吸纳各方面意见和建议。坚持从源头上预防和治理腐败，加大查办大案要案工作力度。坚决纠正行业和部门不正之风。认真贯彻《行政许可法》，积极推进依法行政，强化行政执法监督，再次精简行政审批事项10%。

各位代表，过去一年成绩的取得，是省委、省政府和市委正确领导及市几大班子团结一心、开拓奋进的结果，是人大、政协及各民主党派、群团组织、各界人士和中省直部门监督支持、积极参与的结果，是驻黑河解放军、武警部队无私奉献和全市人民努力奋斗的结果。在此，我代表市政府表示衷心感谢，并致以崇高敬意！

在总结成绩的同时，我们也清醒地看到，发展中还存在诸多困难和问题。主要是经济总量小，结构不合理，经济发展水平与人民群众的期盼仍有较大差距；改革相对滞后，制约和影响经济社会发展的体制和机制障碍还没有完全破除；经济外向度低，旅贸牵动力量较弱，开放型经济格局尚未形成；农业不强，特色、绿色经济仍处于起步阶段，建立市场农业还需时日；立市产业发育慢，工业短腿问题仍然突出；财政拮据状况没有根本改观，财源基础还不坚实；社会保障体系薄弱，就业和再就业压力大，一些群众关注的热点、难点问题解决的不够好，影响社会稳定的因素依然存在。少数政府工作部门和工作人员公仆意识、服务意识不强，作风飘浮、办事效率低，形式主义和奢侈浪费现象时有表现。对这些问题，我们一定高度重视，在今后工作中认真解决。

二、2005年工作安排

2005年是全面完成“十五”计划和谋划“十一五”计划，加快黑河发展至关重要的一年。总体要求是：以邓小平理论和“三个代表”重要思想为指导，牢固树立和认真落实科学发展观，认真贯彻十六届四中全会和省委九届六次全会精神，按照市委三届二次全会部署，深入实施旅贸牵动、工业立市、科教兴市战略，夯实农业基础，加快农业强市进程，抢占对俄经贸科技合作制高点，推动对外开放战略升级，加强项目建设，壮大支柱产业群体，继续深化改革，加快体制和机制创新，促进经济社会全面协调可持续发展，顺利完成“十五”计划，为“十一五”计划奠定坚实基础。预期目标是：生产总值增长8%，其中一二三产分别增长3%、8.5%、11%。固定资产投资增长7%。财政一般预算收入增长3%。社会消费品零售总额增长9%。人口自然增长率控制在4.5‰。围绕上述目标，重点做好八个方面工作：

（一）坚持城乡统筹发展，不断提高农业整体效益。农业增加值增长3%，农民人均纯收入增长4%。深化结构调整。坚持“打绿色牌，走特色路”，推进优势农产品区域化布局，绿色食品种植面积要达到260万亩。坚持粮牧主辅换位，重点推进优质奶牛、肉牛、绒山羊“两区一带”标准化建设，完成农村畜牧服务体系，力争实现畜牧业产值三分天下。林业在进一步加大国家重点生态林管护，加强资源保护、推进生态建设的同时，大力发展林产经济，实现以林养林、以农养林和以牧养林的良性循环。做大做强北安完达山、九三油脂、爱辉铃田、孙吴沙棘等产业化龙头企业，通过订单和农户参股经营实现利益联接。引导农民兴办专业协会、专业合作社等新型农民合作经济组织，提高农民的组织化程度。发展劳务和外向型经济。加强宣传引导、技能培训、市场对接和维权服务，扩大劳务输出，力争转移农村劳动力12.6万人，劳务收入4亿元。大力发展外向型农业，通过政府引导、市场运作、多元投入，境外开发俄土地10万亩，境内建设爱辉、逊克蔬菜出口基地，建设对俄农产品出口交易中心和黑河中俄农业科技示范园区。推进基础设施工程化和农业标准化。大力开展农田水利工程建设，重点搞好抗旱水源、节水设施、水土流失治理和防洪排涝等工程。确保象山水库除险加固工程开工建设。提高人工影响天气的能力。加快大型农机具更新步伐，力争三年内完成更新任务。进一步发展农机合作社，扩大场县共建规模。实施农业标准化工程，建设种植科技示范园区和畜牧养殖小区，种植业优质品种率达95%以上，畜牧优质品种率达85%。营造农业发展良好环境。继续巩固农村税费改革成果，落实中央和省关于扶持农业发展、减轻农民负担的一系列政策规定。推进农村土地经营权流转，实现土地规模化经营。深化粮食流通体制改革，搞活流通服务。推进农村信用社改革，扩大支农信贷资金规模。

（二）培育立市产业群体，壮大工业规模。全市规模以上工业企业增加值增长8%。发育五大支柱产业。矿产资源产业重点引进战略投资者，推进翠宏山铁多金属矿、东安和三道湾子岩金矿、多宝山铜矿的开发，加快推进黑河恒基水泥有限公司项目建设。能源产业集中做好“煤转电”和“水转电”两大支柱工程，加快推进西部金水木耳气、东部西岗子煤田地质补勘等前期工作，为建设嫩江电厂和黑河电厂提供充分的煤炭储量依据。全力支持60万吨兴边煤矿建设，争取年内投产，富宏、东兴煤矿改扩建项目年内开工建设。启动黑河20万吨煤矸石电厂项目前期，开工建设嫩江热电厂和金湾水电站。继续推进乌二、乌三、三湾水电站项目前期工作。绿色、特色食品产业立足资源，突出特色，以山野菜、大豆、马铃薯、乳品肉牛精深加工为重点，加快对现有企业改造升级，扩规模、增品种、创品牌，逐步建立新型食品工业体系。医药产业挖掘三精黑河药业、北安飞龙动物制药等现有企业潜力，进一步提高生产能力。完成三精孙吴儿童大药厂ＧＭＰ认证，早日投产达效。加快北药项目开发，提高医药产业在工业中的比重。林木产业重点扶持联宜、圣中等企业，不断壮大木材生产加工基地规模。推进企业技术升级。重点推进嫩江春酿酒有限公司蚂蚁发酵设备改造、嫩江辰鹰乳业1000吨铁听奶粉设备改造、北安进发塑料帕萨特汽车配件90万件设备改造等9个技改和扩能项目。抓好针状焦、超高功率电极等7个新产品开发项目，提升科技含量。深化国有企业改革。通过引入战略投资者等多种方式，促进黑河市热电厂、黑河地铁等重点企业产权多元化。稳妥推进多宝山铜矿、西岗子煤矿等企业政策性破产重组。对具备改制条件的国有工业小企业实施国有资本退出，集中处置国有企业在工商银行的不良贷款工作。强化工业经济调控和服务。进一步完善重点县（市）、重点行业、重点企业经济运行的监测分析和预测预警体系。搞好综合协调，为重点企业解决产品外运问题。抓好重点亏损大户减亏和盈利大户增盈，促进企业效益与质量同步增长。

（三）以对俄经贸科技战略升级为重点，进一步扩大对外开放。对外贸易进出口总值增长20%，利用外资增长10%。全力推进项目合作。力争黑龙江公路大桥开工建设。尽早建设黑河与布市铁路间油（气）运输换装管道。支持好运木业在腾达和结雅采伐30万立方米木材等在俄森林采伐项目。以华富集团、华兴公司为主体，扩大俄远东地区房地产市场占有份额，拉动建筑材料出口。配合省国土资源厅尽快取得俄加林铁矿独立采矿权。利用对俄地缘优势引进企业在布市创办家电组装厂，依托克市中国商品城建设鞋业组装厂，形成境内境外合成组装的产业链条，实现双向过埠增值。扩大贸易规模。积极运作对俄原油、成品油、天然气和丁基橡胶、聚丙烯、聚乙稀等高附加值化工原料进口；以俄克市、赤塔、下卡姆斯克市等区域为重点，拓展空间，不断扩大边境小额贸易规模。吸引更多国内外客商到黑河从事商品批发、储运与中介业务，扩大民贸规模。加强合作，扩大代理，提高一般贸易进出口额度。壮大经营主体。坚持引进外地大户与培育本地优势企业并重原则，鼓励各类企业参与对外贸易，推进贸易主体多元化。加强资金、政策和服务保障，扶持外经贸大户，力争贸易额超千万美元外经贸企业达到6家。加快园区建设。完成新兴基础原材料园区二期工程基础设施建设，筛选落实入区企业，年内确保对俄购电实现4亿度。进出口加工园区力争和黑龙江大桥建设同步进行。加快吉斯达国际商务港和信用保障体系建设，形成集生产加工、贸易、物流、研发为一体的跨国采购中心。协调俄阿州尽早建立自由经济区。创优口岸环境。年内开工建设黑河口岸货检现场新建工程项目。协调俄方实施客货分流，继续做好开通黑河航空口岸相关工作。推动招商引资再上新台阶。瞄准国内和世界500强企业集团，重点围绕矿产、农林、旅游等开发及对俄经贸合作、企业产权改革和城市建设进行引资、引智、引项目，力争在招商规模和质量上都有大的飞跃，确保引进到位资金总量和超千万元大项目资金额增长20%以上。

（四）加速景区建设和线路开发，推动旅游业快速发展。全年接待国内外旅游者增长8.8%，其中边境游人数增长11%。加快景区景点建设。五大连池景区重点做好火山观光、矿泉疗养、地质科考、休闲度假等系列产品开发，加快格球山天池、温泊开发进度，争取游客接待中心年底建成，完成景区AAAA级质量等级评定。加快黑河龙珠远东国际滑雪场二期工程及水上乐园、民俗风情园等项目建设。推进瑗珲历史文化旅游区建设，完善景点设施，增强吸引力。加快山口电站和宝山电站的工业旅游、嫩江农场和大西江农场的农业旅游示范点开发，力争两年内进入省级示范点行列。强化线路连接。挖掘火山游、边境游特色，通过有效对接，推出一批新的旅游精品线路。加大旅游要素整合力度，延长旅游产业链。加强与国内外重点城市的联系，抓好俄伊尔库茨克、雅库茨克旅游线路的延伸工作。加大宣传促销。强化整体促销意识，深入俄罗斯、国内重点客源城市开展宣传促销。加大在主流媒体上的广告宣传，打造黑河旅游品牌。加强行业管理。倡导公平竞争，推行优质服务。强化市场管理，抓好“诚信之旅在黑河”活动，提高从业人员素质。协调俄方改善接待设施，提高服务质量。采取有力措施，不断扶壮经营主体规模。

（五）加快发展民营经济，增强经济发展活力。民营经济增加值增长15%。落实扶持政策。认真落实中央和省市有关支持民营经济发展的各项政策措施，清除一切歧视性的做法和规定，为民营经济发展创造良好的外部环境，使民营经济平等参与市场竞争。拓宽市场准入领域，积极引导民间资本进入基础设施、公用事业等法律法规未禁入的行业和领域，不断拓展民营经济发展空间。抓好信用担保体系、诚信评价体系、人才培训体系、法律服务体系建设，努力解决融资难问题，为民营企业提供更优良、更直接的服务。扩张经济总量。拓宽发展空间，引导企业通过参股、兼并、购买等形式参与公有制企业产权重组，扩张规模。扶持一批主业突出、管理先进、核心竞争力强的优势企业，重点做大畜牧、绿色、山产品、饮食等20个规模较大、发展前景较好的骨干民营企业和50家民企大户。上缴税金过百万元的民营企业要达到15 家以上。促进结构优化。大力发展高新技术企业和民营科技企业，支持各类科技人员领办、创办民营科技企业，优化企业结构，带动产业升级，力争民营科技企业发展到20家。加快发展农副产品加工和出口创汇型民营企业，帮助更多企业争取自营进出口权。支持民营企业由传统服务业向旅游、信息咨询、物流配送、社区服务等现代服务业拓展。鼓励民营企业开发新产品，优化产品结构，培养具有黑河特色的地方产品，争创省内国内知名品牌。

（六）适应发展需要，推动城市建设上层次、上水平。准确把握城市发展的特点和规律，以规划为龙头，以经营为手段，依法、规范、有序地做好城建工作。强化规划的“龙头”地位和作用。坚持规划和土地集中审定制度，增强规划执行的严肃性、透明性。高层次、高水平编制黑龙江大桥桥头区详控规划。加强规划监察，建立日常检查和巡查制度，依法查处擅自改变建筑立面、色调、间距、结构的建设项目，维护规划法律地位。加快推进城市基础设施建设。根据本地实际，确定城市道路、给排水、供热、绿化等基础设施建设计划，全力抓好组织实施。市区利用好国家和省给予的城市建设资金，抓好城市基础设施完善、改造和新建，完善城市功能，改善城市环境。保持房屋建设持续健康发展。把房屋建设作为扩大需求、拉动各业发展的重要力量，结合平房区、棚户区改造，继续鼓励发展集资建房或合作建房，保持房屋建设稳步增长的势头。完善住房公积金制度，为职工改善住房创造条件。建立高效运行的城市管理体系。健全城市管理责任制，明确管理区域，细化管理责任，严格评定标准及奖惩措施。对城市出入口、重点路段和市容市貌进行有效规范和专项整治。创新管理机制，充分调动社区、街道和社会力量的积极性，加快城市管理市场化步伐。抓好物业管理工作，提高覆盖面和服务水平。有效规范土地、建筑和房地产市场。巩固土地市场治理整顿成果，建立国土资源市场长效管理机制。进一步健全完善招投标制度，杜绝建设过程中的各种违法违规行为，严格落实工程质量终身责任制。规范房地产开发企业的经营行为，加强监督管理，促使公平竞争。依法解决土地征用、房屋拆迁、拖欠工程款问题。坚持不懈抓好生态市建设工作。按照生态市建设规划，制定全市退耕还林、退耕还草、治理水土流失、农村生态环境保护、自然保护区建设的年度计划。抓住国家发展热电联产集中供热的机遇，加快集中供热项目的争取和建设工作。推进 “无烟城市”创建工作，2005年市区并入集中供热10万平方米。全面禁止砂金开采，坚决遏制对生态环境的破坏。稳步推进小城镇建设。抓紧完成市域内部分基础条件好、发展潜力大的建制镇的规划编制工作，科学确定发展定位，为小城镇建设有序进行创造条件。加快小城镇道路、给排水、供电、绿化等设施建设，完善小城镇功能。

（七）加强财税金融工作，发挥投资对经济增长的拉动作用。围绕经济抓财源，上项目，促增收，保证国民经济健康有序运行。确保财政有序高效运转。加强税收征管，加大监督和稽查力度，重点解决税收跑冒偷漏问题，维护税收秩序。切实加强非税收入和国有资产收益管理，强化耕地占用税、契税等税种收缴，增加可用财力。坚持过紧日子思想，合理调整和优化支出结构，重点解决“三农”问题、公共卫生建设、扩大就业、完善社会保障制度，确保养老和工资的发放。结合事业单位改革，清理财政供养人员，减轻财政负担。严格控制车辆配备，压缩会议费、电话费、接待费支出规模，积极探索职务消费货币化的新路子。切实加强财政监督，逐步建立预算绩效评价体系，完善支出管理机制，从源头上、制度上防止铺张浪费。树立新的理财观念，推行综合财政预算，扩大国库集中支付改革范围，深化政府采购改革，加大非税收入管理改革力度，提高财政管理水平。加强政府债务管理，努力防范和化解财政风险。千方百计扩大投资需求。各地各部门要认真总结“十五”计划，做好“十一五”计划编制工作，用规划指导和推动项目建设。认真研究国家和省有关政策，集中力量建设一批对经济发展有重大带动作用的项目。积极运作地铁划归国铁运营工作，争取完成111国道嫩江至讷河前期工作，实现省道嫩江至多宝山段、嫩江至黑河段开工建设，完成黑河国际货运中心前期工作及征地拆迁工作。加强项目储备工作，根据国家投资方向、投资领域的新变化和国内外资本流动的新动向，高起点、高标准地储备一批新项目、大项目，防止低水平重复建设。加强地方金融监管。保持金融平稳运行。规范发展资本市场，全面改进金融服务，加大对经济发展的支持力度。加强同金融部门的沟通与衔接，积极推荐优质项目，配合处理不良债务，扩大利用银行信贷投资。

（八）坚持以人为本，努力构建和谐社会。推进三个文明同步发展，搞好社会安全稳定，实现经济社会全面进步。协调发展各项社会事业。发挥科技和人才对经济社会发展的支撑作用。推进经济社会信息化工程，做好知识产权和科技成果的保护和管理工作。加强对俄科技合作，搭建中俄科技交流合作平台。优先发展教育。巩固“普九”成果，保证适龄人口接受义务教育。完善教育信息化和远程教育工程，完成中小学危房改造计划，严格实施中小学“一费制”。推进教育人事制度改革，扩大课程改革实验范围，努力提高教学质量。支持民办教育和合作办学，拓宽教育发民途径。扩大职业教育社会覆盖面，培养实用型职业技术人才。办好黑河学院，努力向规范化本科院校迈进。进一步完善公共卫生体系建设，不断提高应对突发公共卫生事件的能力。强化农村卫生工作，加大乡镇卫生院基础设施投入。做好新型农村合作医疗试点，扩大社区卫生服务覆盖面。加大食品市场的管理和整顿工作，保障人民群众的食品安全。抓好人口与计划生育工作，实现省下达的人口与计划生育控制目标。扎实推进“边疆文化名城”建设，筹建“市区群众文化活动中心”，确保市中心图书馆建成使用。实施有线电视“南三县”光缆化改造，加快城乡联网步伐，积极推进黑河有线电视数字化。进一步做好其他社会工作。做好就业和社会保障工作。千方百计创造就业岗位，倡导和推广灵活多样的就业方式，全年城镇登记失业率控制在5.5%以内。全面推进社会保障试点工作，争取完成4.7万人的并轨任务。完善城镇职工基本养老保险、失业保险和城镇居民最低生活保障制度，加快推进医疗保险和工伤保险制度，依法扩大各项社会保险覆盖面，切实做到应保尽保。进一步加大征缴力度，多渠道筹集和管好社会保障基金，确保各项参保待遇按时足额发放。建立健全最低工资保障制度。完善城乡社会救助体系。切实加大地方救灾、社会救助方面的投入，适时开展农村最低生活保障试点工作，深入实施农村特困医疗救助制度，加快以医疗、教育救助为主要内容的城市综合社会救助体系建设，倡导社会捐助，依法保障城乡困难群众获得基本物质帮助的权益。维护社会安全稳定。加强信访工作，建立和完善长效机制，集中化解信访积案，积极预防和及时处理新的信访案件。建立健全预警和快速反应机制，有效预防、及时处置群体性事件。加强社会治安综合治理，继续开展打黑除恶斗争，加大案件侦破力度。全面落实安全生产责任制，加大专项整治力度，避免各类重特大安全事故发生。

三、加强政府自身建设，全面提高施政能力

今年全市经济社会发展的任务十分繁重，各级政府一定要审时度势，抢抓机遇，知难而进，奋发图强，开创各项工作的新局面。

解放思想，振奋精神。黑河经济欠发达，根本原因之一在于思想不够解放，精神状态欠佳，进取心不足。面对加快发展的时代要求和黑河的现实压力，我们一定要突出解放思想的主旋律。以思想的解放为统领，抓机遇、创思路、变方法，谋求加快发展的新途径。重点要在学习领会和贯彻上级精神上解放思想，一切围绕快发展、大发展来贯彻执行，大胆寻找政策的杠杆，撬开发展缝隙。在创新发展思路上解放思想，从破解难题切入，抓优势带劣势，变守业思路为创业思路，创造发展的新业绩。在改进方式方法上解放思想，不断研究新情况，把握新特点，只要有利于快发展、大发展，就要突破定势，大胆实践，开辟新途径，解决新问题。在解放思想的同时，更要振奋精神。不能因黑河的暂时落后而萎靡不振，裹足不前，而是要正视困难，振奋精神，放开手脚，迎难而上，以更加坚定的信念，更加高昂的斗志，更加饱满的热情，投入到各项工作中去。

坚持民主、科学、依法行政，健全政府运行机制。主动接受市委领导，认真执行各项决议、决定。推进民主行政，完善与市委、人大政协和社会各界的沟通机制，自觉接受人大和政协监督，及时办理议案、建议和提案，定期报告工作，通报情况。认真听取工会、共青团、妇联等群众团体的意见。推进科学行政，不断完善决策机制，努力提高科学决策水平和公众参与度。全面贯彻《行政许可法》，以落实和完善行政执法责任制、评议考核和过错追究制为重点，推进依法行政。加大层级监督力度，解决多头执法和越权执法问题，努力建设法治政府。

转变政府职能，创新服务方式。继续推进政企、政事、政社分开，切实将政府职能转变到经济调节、市场监管、社会管理和公共服务上来。狠抓软环境治理，严厉惩处破坏环境的人和事，营造公正公平的法制环境、公开透明的政策环境、竞争有序的市场环境和优质高效的服务环境。深化政务公开，扩大公开范围，突出公开重点，健全长效机制，增强政府工作的透明度。不断拓展政务大厅的服务领域，凡涉及行政审批的事项都要在政务大厅办理。尽快建立新闻发言人制度，及时公布政务信息，维护群众的知情权。加快推行电子政务，加快工作节奏，提高办事效率，增强行政活力。严格执行“六项制度”，畅通市长热线电话，建立市领导与客商交流的绿色通道，切实为企业排忧解难，不断提高服务水平。

改进工作作风，狠抓工作落实。按照为民、务实的要求，进一步转变工作作风，讲实话、办实事、求实绩，工作上既积极进取，又量力而行，不盲目攀比，不虚报浮夸，不急功近利，不搞劳民伤财的“形象工程”和“政绩工程”。建立健全抓落实的工作机制，明确责任，强化督查，严格奖惩，根治“中梗阻”和“末稍炎”，确保政令畅通，落实到位。减少会议和文件，集中时间和精力，深入到基层企业、落后农村、社区和街巷，深入贫困群众和弱势群体，体察民情，了解他们的困难和需求。强化宗旨观念和公仆意识，以真心实意为人民群众负责的态度，凡是涉及群众利益的事情，再小也要竭尽全力办好、办实，切实做到施政为民。

廉洁自律，从严治政。加强对政府工作人员警示教育，深入开展“四承诺”、“三树立”和“两禁止”活动，树立正确的人生观、世界观、价值观和权力观、地位观、利益观。坚持从源头上预防和治理腐败，以“制权、管钱、用人”为主线，完善重大事项报告制度和民主评议制度，建立结构合理、配置科学、程序严密、制约有效的权利运行机制。发挥行政监察和审计机关的职能作用，依法查处贪污受贿、以权谋私和侵害群众利益的违纪违法案件。全面落实党风廉政建设责任制，建立科学的激励机制和考核指标体系，树立政府为民、廉洁、务实的良好形象。

各位代表！全面建设小康社会需要我们奋力开拓，完成今年各项任务需要我们共同拼搏。让我们高举邓小平理论和“三个代表”重要思想伟大旗帜，在市委的坚强领导下，依靠和带领全市人民，与时俱进，开拓创新，同心同德，真抓实干，为加快发展、富民强市、建设新型开放城市做出新的更大的贡献！
